# New Insights into the Role of Cellular Senescence and Its Therapeutic Implications in Ocular Diseases

**DOI:** 10.3390/bioengineering12060563

**Published:** 2025-05-23

**Authors:** Junying Wu, Xiuxing Liu, Yidan Liu, Wenru Su, Yehong Zhuo

**Affiliations:** 1 State Key Laboratory of Ophthalmology, Zhongshan Ophthalmic Center, Sun Yat-sen University, Guangdong Provincial Key Laboratory of Ophthalmology and Visual Science, Guangzhou 510060, Chinaliuxx65@mail2.sysu.edu.cn (X.L.);; 2 Department of Ophthalmology, Ninth People’s Hospital, Shanghai Jiao Tong University School of Medicine, Shanghai 200011, China

**Keywords:** senescent cells, senescence-associated secretory phenotypes, ocular disease, senolytics, epigenetic reprogramming, oxidative stress

## Abstract

The process of aging exerts profound effects on various physiological systems, leading to the progression of chronic degenerative disorders and pathologies associated with advancing age. Cellular senescence plays a central role in the aging process and the onset of various eye conditions associated with advancing age, including age-related macular degeneration (AMD), diabetic retinopathy (DR), glaucoma, cataracts, and ocular surface disorders. The accumulation of senescent cells (SnCs) and their secretion of pro-inflammatory and tissue-remodeling factors, collectively known as the senescence-associated secretory phenotype (SASP), exacerbate chronic inflammation, oxidative stress, and tissue dysfunction, contributing to disease progression. This study is the first to systematically integrate the multifaceted mechanisms of cellular senescence in ocular diseases, revealing differential regulatory mechanisms of specific signaling pathways across different ocular pathologies, thereby providing novel insights into the pathogenesis of these disorders. SnC-targeted therapies such as senolytics, senomorphics, SASP modulators, mitochondrial-targeted antioxidants, and epigenetic reprogramming are emerging as regenerative therapies, demonstrating potent anti-inflammatory effects, restoration of normal tissue physiology, and successful regeneration of ocular defects in preclinical models and clinical trials, while slowing senescence-associated disease progression. This review not only summarizes the role of cellular senescence in ocular diseases but also delves into potential therapeutic strategies, particularly highlighting novel perspectives for root-cause-targeted therapies from the unique angle of senescence biology, which may pioneer new directions for the treatment of ocular pathologies.

## 1. Introduction

Aging represents a certain and universal biological process that progressively compromises the functional integrity of nearly every physiological system, creating a cumulative burden that fundamentally reshapes human health trajectories. Similarly to other organs, the eye experiences functional decline with age, a condition that has become increasingly common in the growing aging population. Cellular senescence represents a fundamental biological mechanism marked by the permanent arrest of cell proliferation, typically associated with the emergence of a distinctive secretory profile that releases inflammatory mediators and extracellular matrix-modulating components, collectively termed the SASP [1,2]. Initially, cellular senescence was recognized as a protective mechanism to limit uncontrolled cell proliferation and prevent tumorigenesis. However, increasing evidence has revealed that the accumulation of SnCs is closely linked to the onset and progression of various age-related diseases, including ocular disorders [3]. Through the secretion of pro-inflammatory factors, cytokines, and other SASP components, SnCs contribute to chronic inflammation and exert paracrine effects on neighboring tissues, ultimately driving disease progression.

In the field of ophthalmology, SnCs have garnered increasing attention for their roles in AMD, DR, glaucoma, cataracts, and ocular surface disorders. Studies have shown that the development and progression of these diseases are closely associated with cellular senescence and its pathological processes. For instance, SnCs accelerate oxidative stress, disrupt extracellular matrix homeostasis, and impair tissue repair mechanisms, directly or indirectly contributing to ocular disease pathology. Moreover, the pleiotropic effects of SASP can play dual roles, offering reparative benefits in the early stages of disease while driving chronic inflammation, tissue degradation, and cell death in later stages.

In recent years, therapeutic strategies aimed at eliminating senescent cells or modulating their activity have emerged as promising approaches for the treatment of age-related ocular diseases, as demonstrated by advances in nanomedicine-based senolytic delivery systems [4]. These include senolytic agents that selectively eliminate SnCs and SASP-modulating therapies that aim to mitigate their harmful effects [5]. These innovative approaches not only have the potential to slow disease progression but may also promote tissue repair and functional recovery.

This review seeks to offer an in-depth overview of the defining features, biological sources, and functional roles of SnCs as well as their linked SASP. It will also focus on the roles of cellular senescence in AMD, DR, glaucoma, cataracts, and ocular surface disorders, exploring the underlying mechanisms. Furthermore, we will examine SnC-targeted therapeutic strategies, including senolytics, senomorphics, and molecular pathway modulators, which act as regenerative therapies by suppressing inflammation, restoring tissue physiology, and promoting ocular defect regeneration. These approaches also provide novel perspectives for elucidating senescence–ocular health interactions and advancing clinical interventions targeting age-related eye disorders.

## 2. Characteristics of SnCs

**Figure 1:**
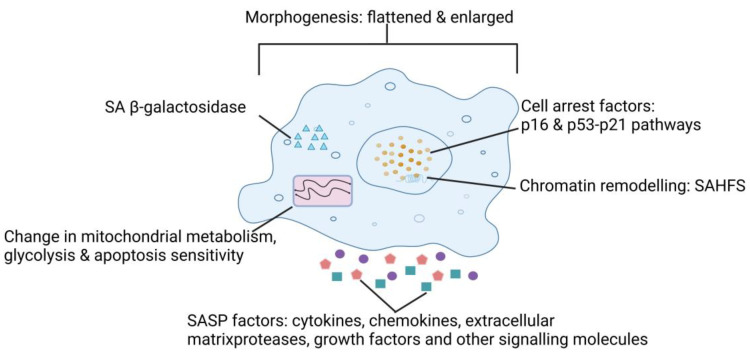
Characteristics of senescent cells. This diagram vividly illustrates the characteristic phenotypic alterations linked to cellular senescence. Morphologically, they are flattened and enlarged. SA β-galactosidase is a biochemical marker for identification. Cell cycle arrest is due to p16 and p53-p21 pathways, and chromatin forms SAHFS. There are metabolic changes in mitochondria, glycolysis, and apoptosis sensitivity. Moreover, senescent cells secrete SASP factors like cytokines, chemokines, etc.

Cellular senescence, first identified by Hayflick and Moorhead in 1961, is a crucial mechanism that determines cell fate and is recognized as a key feature of aging [6]. Modern perspectives characterize senescence as a dynamic, context-dependent process wherein SnCs undergo continuous phenotypic evolution, transitioning through distinct functional states [1,7]. These cells exhibit hallmark features including irreversible G1-phase cell cycle arrest, cytomorphological remodeling (hypertrophic morphology, multinuclearity, and nuclear envelope expansion), lysosomal hyperactivation marked by SA-β-Gal activity, macromolecular damage accrual, and secretion of a pleiotropic SASP (Figure 1) [2,8,9]. Cellular senescence programs, activated by diverse intrinsic/extrinsic stressors including genomic instability and metabolic perturbations (Table 1), converge through coordinated DNA damage response (DDR) pathways and epigenetic remodeling [5]. These molecular events culminate in ultimately activating two core tumor suppressor axes: the p53-p21CIP1 genomic surveillance network and the p16INK4A-RB cell cycle termination module [10]. This mechanistic framework underlies SnCs’ dual roles in maintaining tissue homeostasis and driving age-related pathologies.

**Figure 2:**
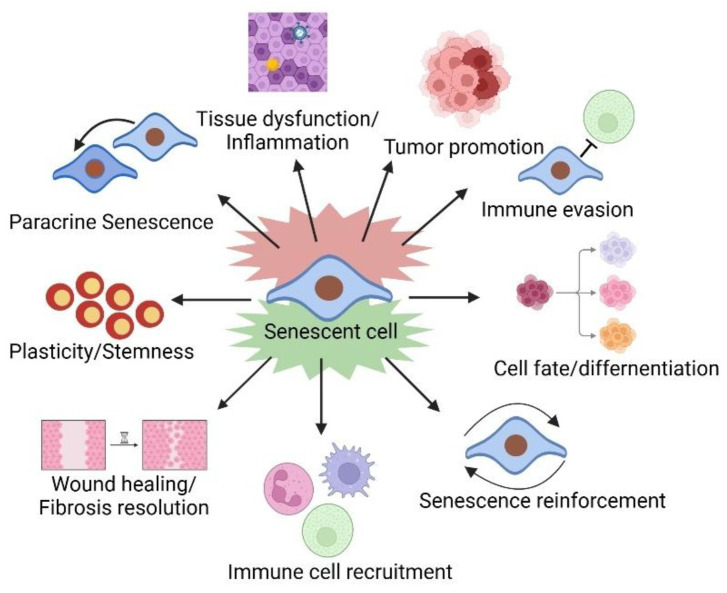
The diverse functions of the SASP. This diagram summarizes the effects mediated by senescent cells (center) through the SASP. The effects depicted above the senescent cell (in red) highlight the detrimental consequences of the SASP, while those below (in green) represent its beneficial effects.

Senescence was initially considered a phenomenon specific to tissue culture conditions. However, later studies have underscored the significance of senescence in both pathological and physiological processes (Figure 2). Senescence plays pivotal physiological roles in normal development [11,12,13], the maintenance of tissue homeostasis, and the regulation of tissue remodeling and repair [14]. It also plays a role in wound healing [15,16] and the production and release of insulin from pancreatic β-cells [17]. Additionally, it curbs tumor progression by preventing dysfunctional, damaged, or transformed cells from transmitting their genomes to future generations [18,19,20,21,22,23]. Cellular senescence demonstrates context-dependent duality, serving transient protective functions in developmental morphogenesis, wound healing, and tumor suppression [24], whereas its chronic persistence becomes pathologically consequential through promoting organismal aging and senescence-associated disorders [25].

To precisely regulate the dual nature of cellular senescence (pathological vs. protective), recent studies have emphasized decoding the “senescence code”, which involves cell-type-specific epigenetic–metabolic interactions to shape the spatiotemporal dynamics of the SASP. This approach is particularly relevant to ocular diseases. In AMD, for example, senescent retinal pigment epithelial (RPE) cells and their SASP profile contribute to inflammation and disease progression. Future strategies may include CRISPR-based modulation of SASP, phase-specific interventions targeting transitional senescence to prevent irreversible cell aging, and organelle-specific senolysis exploiting mitochondrial–lysosomal dependencies for treating inflammatory eye diseases such as uveitis. These approaches may help reprogram pathological senescence while preserving its physiological functions, offering new therapeutic opportunities for ocular diseases.

## 3. Brief Introduction and Classification of SASP

Numerous studies have demonstrated that SnCs secrete a variety of bioactive molecules, such as cytokines, chemokines, metalloproteases, and growth factors, as components of the SASP. These molecules exert their effects via autocrine and paracrine signaling mechanisms, influencing both the SnCs themselves and adjacent cell populations [26].

Based on their mechanisms of action, SASP factors can be classified into the following categories [27]: (1) receptor-mediated, encompassing growth factors (VEGF, FGF, HGF, TGF-β, and GM-CSF), chemokines (GROα, GROβ, CCL-2, CCL-5, CCL-16, CCL-26, and CCL-20), and interleukins (IL-6, IL-8, and IL-1α) [28]; (2) regulatory molecules, including insulin-like growth factor-binding proteins (IGFBP), the plasminogen activator inhibitor (PAI), and tissue inhibitors of metalloproteases (TIMPs) [29]; (3) directly acting molecules, including matrix metalloproteases (MMP-1, MMP-10, and MMP-3) and serine proteases such as the tissue plasminogen activator (tPA) and urokinase plasminogen activator (uPA) [29]. Several in vitro and in vivo studies have attributed the multifunctional effects of the SASP to individual protein components [29]. For example, SASP components like IL-6, IL-8, and CCL2 contribute to tumor cell growth [30,31]. VEGF stimulates vasculogenesis, and PAI-1, IGFBP7, IL-6, and IL-8 contribute to the enhancement of cellular senescence [28,30,32,33]. Studies have suggested that TGF-β family ligands, VEGF, and CCL2 contribute to cellular senescence [34], whereas PDGF-AA facilitates wound healing [16,35].

In SnCs, transcriptional activation enhances the expression of numerous previously mentioned SASP components [28,32,36]. Nevertheless, numerous bioactive components of the SASP can trigger inflammatory responses, impair tissue organization, and facilitate the progression of cancerous changes [37]. Notably, blocking SASP factors like IL-6 only partially attenuates paracrine senescence, indicating the presence of additional signaling pathways [38,39]. Evidence increasingly suggests that extracellular vesicles (EVs) secreted by SnCs exhibit unique properties and contribute to modulating the behavior of target cells, much like SASP bioactive molecules [40,41,42,43,44]. Although the precise mechanisms remain to be fully elucidated, senescence-associated EVs may act as a parallel signaling route to soluble SASP factors, especially in the context of long-range intercellular communication. Thus, EVs released by SnCs, referred to as senescence-associated EVs, represent a newly identified component of the SASP [45,46,47].

## 4. The Pleiotropic Effects of the SASP

The SASP, a hallmark of SnCs, mediates intercellular communication through regulated secretion of bioactive factors comprising immunomodulators (IL-6/IL-8), protease inhibitors (PAI-1), metabolic regulators (IGFBP7), and extracellular matrix modifiers. This signaling cascade executes dual functions: (1) promoting SnCs clearance via chemokine-driven phagocyte recruitment while reinforcing growth arrest through autocrine CDKN1A/p21 activation; and (2) inducing paracrine senescence in neighboring cells through TGF-β ligands, VEGF, and monocyte chemoattractants (CCL2/CCL20), thereby establishing senescence-permissive microenvironments. Mechanistic studies have confirmed that SASP components sustain cell cycle exit in vitro and exert context-dependent tumor-suppressive or pathogenic effects in vivo [28,34,48].

On the one hand, the SASP plays a pivotal role in orchestrating numerous additional benefits that are intricately linked with the process of acute senescence (Figure 1). The SASP plays a crucial role in its capacity to transmit signals to a variety of immune cells, such as macrophages, T cells, and natural killer (NK) cells [2]. Senescent fibroblasts play a pivotal role in the process of wound healing [16]. In response to tissue damage, the SASP exhibits the capability to facilitate cellular reprogramming within adjacent cells [49]. Furthermore, the SASP enhances plasticity and stemness characteristics of these neighboring cells, thereby contributing to the overall regenerative process [50]. These regenerative effects imply that short-term activation of the SASP may be beneficial in contexts such as tissue repair or organogenesis, which should be considered when designing senescence-targeting therapies to avoid disrupting physiological repair mechanisms. The SASP may also help explain chemotherapy side effects, including bone marrow suppression, cardiac dysfunction, blood clotting, and cancer relapse [51,52]. Recently, it has come to light that SnCs fleetingly arise during the development of mammalian organs [53]. Within this context, SASP factors play a pivotal role in eliciting the differentiation of adjacent cells and facilitating the elimination of superfluous cells during the developmental process [53]. Therefore, SASP factors possess the remarkable capability to orchestrate cell fate reprogramming [11,12,16,49,54,55]. These findings underscore the temporally restricted and highly regulated nature of the physiological SASP, which contrasts starkly with its persistent activation in aging and disease.

Although SnCs have many beneficial effects, we should pay more attention to their adverse effects. The SASP has been shown to promote epithelial-to-mesenchymal transition (EMT) and facilitate tumor vascularization, indicating its predominantly protumorigenic nature [56]. For instance, the SASP released by preneoplastic hepatocytes can attract immature myeloid cells, which subsequently suppress NK cell activity, ultimately facilitating the development of hepatocellular carcinoma (HCC) [57]. The chronic low-grade inflammation characteristic of aging organisms, clinically designated as inflammaging (inflammation-associated aging), serves as a critical pathophysiological driver in the onset of geriatric comorbidities through sustained immune–metabolic dysregulation [58]. Eliminating SnCs reduces pro-inflammatory cytokine levels in aged mice [59,60], suggesting that the SASP may also play a part in inflammaging. Intriguingly, the implantation of even a minute quantity of SnCs induces physical malfunctions and sufficiently elevates systemic inflammation [61]. The SASP may also play a role in the pathogenesis of disease by disrupting the delicate balance of tissue homeostasis. It may induce alterations in lipid metabolism and modifications in skin phenotype. These actions can lead to changes, including skin phenotypes and lipodystrophy frequently observed in aging mice, further illustrating its complex and multifaceted impact on biological processes [62]. Senescent dendritic cells display upregulated interferon-stimulated gene signatures alongside impaired antigen presentation, while single-cell receptor profiling demonstrates age-related remodeling of CD8+ T cell immunity characterized by contracted clonal diversity and expanded effector, cytotoxic, exhausted, and age-associated B cell (ABC) populations [63].

The pleiotropic nature and paradoxical manifestations of the SASP are dynamically regulated by multiple determinants encompassing senescence-inducing stimuli, cellular lineage specificity, and microenvironmental conditioning [2]. This variability influences the role of senescence in different ocular diseases. In AMD, senescent RPE cells secrete VEGF, IL-6, and complement proteins, promoting inflammation and neovascularization [64]. In glaucoma, senescent trabecular meshwork cells release TGF-β and MMP-9, contributing to impaired aqueous humor outflow [65]. In dry eye disease (DED), senescent corneal and conjunctival cells produce IL-1β, IL-8, and MMP-3, disrupting barrier integrity and sustaining inflammation [66]. These cell-type-specific SASP profiles underscore the importance of context in understanding senescence-associated pathology in the eye and highlight the need for targeted, disease-specific therapeutic strategies. Taken together, the dynamic and context-dependent nature of the SASP suggests that its therapeutic manipulation must be precisely timed and tissue-specific. Further mechanistic studies are needed to elucidate how distinct senescence-inducing pathways shape SASP composition across different ocular cell types and to develop interventions that mitigate its deleterious effects while preserving physiological benefits.

## 5. Regulation of the SASP

**Figure 3:**
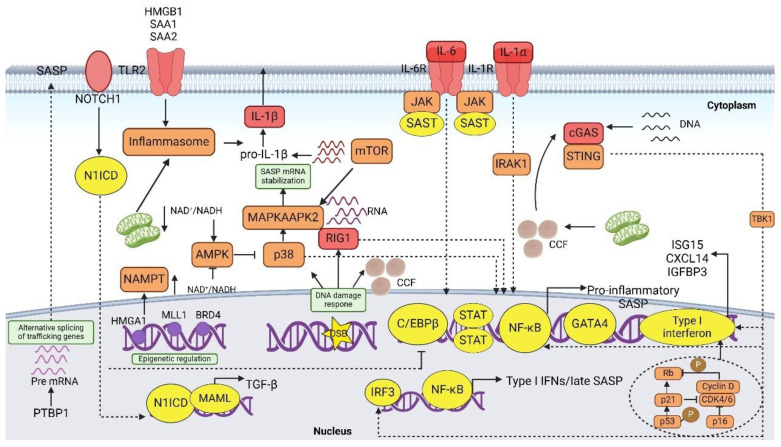
Regulation of the SASP. The schematic illustrates the various factors involved in SASP induction. Sensors, receptors, and ligands are highlighted in red, intracellular signaling components in orange, and transcription factors in yellow. Abbreviations: (CCF) chromatin cytoplasmic foci, (DSB) double-strand break.

Next, we summarize the underlying mechanisms that govern its regulation. Although the precise triggers that activate the SASP remain elusive, they are often intricately linked to the DNA damage response (DDR) and converge in a concerted manner to elicit a transcriptional program indispensable for the induction of SASP [2]. However, it has been demonstrated that several alternative pathways, independent of DDR, are capable of activating different classes of SASP proteins [67]. Additionally, alterations in lipid homeostasis pathways have been observed in cellular senescence, serving as valuable markers for identifying lipid constituents of the SASP [68]. SnCs also exhibit distinct regulatory patterns in their extracellular vesicle secretion machinery, leading to significant alterations in vesicle release dynamics [69,70]. This section focuses on elucidating the key molecular pathways that control SASP protein expression, including their interconnections and regulatory networks (Figure 3).

### 5.1. SASP Induction

SnCs activate SASP through innate immune sensors detecting molecular danger signals [2]. Key sensors like RIG-I, which detects cytoplasmic RNA, and the inflammasome, which recognizes damage-associated molecular patterns (DAMPs), are crucial in mediating SASP-related inflammation [34]. The NLRP3 inflammasome, in particular, contributes to SASP induction by activating inflammatory cascades or pyroptosis [71]. Moreover, cytosolic DNA, such as mitochondrial DNA and cytoplasmic chromatin fragments (CCFs), acts as a key signaling molecule to activate the SASP through the cyclic GMP-AMP synthase–stimulator of interferon genes (cGAS–STING) pathway [72,73]. This pathway activates TBK1, which in turn triggers IRF3 and NF-κB, promoting inflammatory responses [2]. Persistent DDR and mitochondrial dysfunction further enhance SASP, partly by generating cytosolic CCFs [74]. Other factors, such as changes in metabolic pathways (e.g., NAD+/NADH ratios and NAMPT activity), also modulate SASP, linking metabolic shifts to inflammation [62]. Collectively, these signals coordinate the activation of a transcriptional program required for SASP induction, which plays a critical role in the progression of senescence and age-related diseases.

### 5.2. Signaling Pathway

**The DDR and NF-κB pathways.** NF-κB acts as a key regulator of the SASP, orchestrating the expression of interleukins, chemokines, growth factors, and other inflammatory mediators to shape the senescent microenvironment [36,75]. Its functional deficiency disrupts autocrine signaling circuits mediated by IL-6 and IL-8, thereby bypassing senescence-associated proliferative arrest [36,76,77]. Within the SASP regulatory network, the DNA repair protein Polymerase 1 (PARP1) forms a cascade activation axis with ATM kinase, priming NF-κB signaling through IKK complex activation prior to its initiation [30,78]. IκBζ, a selective NF-κB co-activator, specifically regulates key SASP components such as IL-6 and IL-8 [79]. Meanwhile, the transcription factor C/EBPβ collaborates with NF-κB to coordinate the transcriptional program of CXCR2 and pro-inflammatory SASP genes, while the oxidative stress sensor Protein kinase D (PKD) fine-tunes IL-6/IL-8 expression via direct interaction with NF-κB [48,80,81].

Cell surface receptors CD36 [82] and CD40L [83] act as molecular interfaces, transducing senescence-inducing signals to activate the NF-κB-SASP cascade. Notably, DDR components including ATM-CHK2, ATR, and GATA4 synergize with NF-κB to amplify pro-inflammatory SASP production, establishing a multifaceted regulatory framework [18,36,84].

**The p53–p21 and p16–Rb pathways.** SnCs are centrally characterized by irreversible proliferative arrest, synergistically regulated by the p53-p21 and p16-pRB signaling axes, which are concurrently involved in the fine-tuning of the SASP [85]. In p53-deficient hepatocellular carcinoma models, reconstitution of p53 function not only induces cellular senescence but also generates a secretory profile with immune-activating properties [86]. As a key regulatory hub, p53 maintains homeostasis by inhibiting pro-inflammatory SASP factors, and its loss of function leads to aberrant accumulation of inflammatory factors in fibroblasts from a variety of senescence models (replicative, treatment-induced, and oncogene-induced) [31,87]. The MDM2 inhibitor, nutlin-3a, significantly reduces the SASP in TIS cells by stabilizing p53 protein levels [31,87]. Within the MiDAS (Mitotic Catastrophe-Induced Senescence) model framework, genetic ablation of p53 significantly upregulates pro-inflammatory cytokines including IL-1α and IL-1β, whereas chronic nutlin-3a administration promotes cellular senescence while maintaining SASP quiescence [67,88]. Notably, the DDR non-dependent senescence state established by p16 overexpression or long-term CDK4/6 inhibition exhibits a unique p53-dependent secretion profile (p53-arm), containing p53 direct target gene products such as IGFBP3 and interferon pathway-associated factors such as ISG15, despite the lack of typical inflammatory factors [67,89].

Analyzed at the level of the regulatory network, p21 activates immune surveillance functions via Rb-dependent mechanisms by promoting the expression of early secreted factors such as CXCL14 and IGFBP3 [90]. And p16, in addition to mediating cell cycle block, is an essential regulatory element for SASP expression in OIS and treatment-induced senescence models [91]. This multidimensional regulatory property reveals the pivotal position of p16 in the SASP network—both involved in the expression control of classical inflammatory factors and coordinating atypical secretory programs through the p53-arm mechanism—and demonstrates its complex biological functions in shaping the aging microenvironment.

**The p38 MAPK and mTOR pathways.** During tumorigenesis, oncogenic RAS induces premature senescence through sustained activation of the p38 MAPK signaling cascade and its downstream effector molecule, MEK, induces premature senescence by modulating the p53/p16 tumor suppressor pathway [74]. Notably, p38 MAPK is the first key regulator found to directly activate the NF-kB-mediated SASP independently of the DDR [74]. Senescent fibroblasts in the tumor microenvironment maintain SASP factor expression through a p38 MAPK-mediated mRNA stabilization mechanism, which in turn promotes malignant progression [92]. After chemotherapy treatment, the ATM kinase-activated TAK1-MAP3K7 complex drives SASP production by up-regulating the transcription factor ZSCAN4, forming a TAK1-ZSCAN4 regulatory axis that synergistically activates the p38 MAPK/mTOR signaling network [93].

The mTOR signaling pathway amplifies the pro-inflammatory SASP through a dual regulatory axis: (1) by enhancing the biogenesis of membrane-anchored IL-1α and potentiating NF-κB transcriptional activation [39]; and (2) via MK2-mediated phosphorylation that incapacitates the mRNA decay regulator ZFP36L1, establishing a self-reinforcing circuit that stabilizes SASP transcripts (e.g., IL-1α) and amplifies inflammatory signaling through post-transcriptional control mechanisms [38,39]. In line with this mechanism, the mTOR inhibitor rapamycin has been demonstrated to effectively suppress the pro-inflammatory SASP [38,39]. AMP-activated protein kinase (AMPK) antagonizes deleterious SASP secretion by inhibiting mTOR activity under physiological hypoxic conditions [94]. The JAK-STAT pathway, with which it forms a regulatory network interplay, synergistically promotes pro-inflammatory SASP expression and participates in senescence-related functional decline by cross-talking with p38 MAPK/mTOR signaling [95].

**The C/EBPβ pathway.** The CCAAT/enhancer-binding protein (C/EBP) family serves as critical modulators of SASP gene expression, exhibiting dual regulatory capacities in both activation and repression pathways [96]. Structurally conserved across members, these transcription factors contain four functional domains: DNA-binding domain (DBD), a transactivation domain (TAD), leucine zipper dimerization interface, and regulatory domain [97]. Functional antagonism exists between C/EBPβ and C/EBPγ isoforms, with C/EBPβ driving SASP activation through DBD-mediated binding to promoters of inflammatory mediators (IL-1β, IL-6, IL-8, CXCL1, and CXCL7) [97,98], while C/EBPγ heterodimers counteract senescence progression and suppress SASP transcription [99].

Mechanistic studies have revealed that C/EBPβ homodimers are indispensable for RAS-induced premature senescence, whereas C/EBPβ:C/EBPγ heterodimerization creates a dominant-negative complex that inhibits senescence-associated growth arrest [99]. Structural analyses have demonstrated that C/EBPγ lacks functional TAD and forms unstable homodimers, with its depletion triggering tumor cell senescence and proliferation defects [99]. Crucially, genetic compensation experiments have confirmed C/EBPγ’s pro-growth function—C/EBPβ knockout rescues the proliferative impairment in C/EBPγ-deficient cells, establishing C/EBPγ as both an oncogenic driver and senescence suppressor [99].

**The NOTCH1 pathway**. The evolutionarily conserved NOTCH1 signaling pathway exerts temporal control over SASP dynamics through C/EBPβ regulation. During early-phase radiation-induced senescence (RIS), NOTCH1 upregulation directly suppresses C/EBPβ transcriptional activity, potentiating TGF-β1 and TGF-β3 expression in SnCs. This inhibitory effect reverses during later RIS stages, where diminished NOTCH1 activity permits C/EBPβ-mediated induction of pro-inflammatory cytokines. Mechanistic hierarchy analyses have further identified IL-1α as a master upstream regulator that activates C/EBPβ to amplify downstream SASP components. These phased interactions establish NOTCH1 as a bidirectional modulator of SASP programming, coordinating both TGF-β-dominated early responses and cytokine-driven chronic inflammation via C/EBPβ inhibition [77,100,101].

**The JAK-STAT pathway**. The JAK/STAT (Janus kinase/signal transducer and activator of transcription) pathway is a crucial regulator of numerous biological processes, including immune responses, tissue regeneration, hematopoiesis, apoptosis, inflammation, and adipogenesis [102]. This signaling cascade comprises four JAK kinases—JAK1, JAK2, JAK3, and TYK2—that exert their effects through downstream STAT proteins, namely STAT1, STAT2, STAT3, STAT4, STAT5a, STAT5b, and STAT6 [103]. Notably, JAK-STAT signaling exhibits significantly higher activity in SnCs compared to their non-senescent counterparts [103]. The accumulation of senescent preadipocytes in aging adipose tissue fosters the development of a pro-inflammatory SASP, thereby amplifying local inflammatory responses within the adipose microenvironment [95].

Xu et al. conducted mechanistic investigations into the JAK/STAT cascade in age-associated inflammatory processes, demonstrating that pharmacological attenuation of this pathway significantly diminishes SASP factor secretion in both senescent preadipocyte cultures and primary human umbilical vein endothelial cells (HUVECs) [95]. Equally, a study by Chen et al. highlighted that aging leads to the accumulation of senescent tendon stem/progenitor cells (TSPCs) within tendon tissue, with elevated JAK-STAT activity playing a pivotal role in this process [104]. Their study also showed that inhibiting the JAK-STAT pathway with AG490 alleviated senescence-related traits and restored essential functions, including self-renewal, migration, stemness, and actin cytoskeleton dynamics [104].

Beyond its role in cellular senescence and inflammation, JAK-STAT signaling has also been implicated in the pathophysiology of severe COVID-19, particularly in the development of cytokine release syndrome [105,106]. These findings collectively underscore the central role of JAK-STAT signaling in aging-related inflammation and disease pathology, highlighting its potential as a therapeutic target.

**The cytosolic DNA–cGAS–STING pathway.** The cGAS–STING signaling axis emerges as a pivotal regulator of SASP through interferon-mediated inflammatory programming [107,108,109]. Reduced activity of DNA-degrading enzymes, DNASE2 and TREX1, in SnCs allows cytoplasmic DNA to accumulate, triggering cGAS–STING signaling through persistent genomic debris [110]. This process is facilitated by TOP1 cleavage complexes (TOP1cc) and G3BP1, which enhance cGAS recognition and binding to CCFs—a critical step for SASP induction [111,112]. Functional blockade of cGAS or the STING markedly attenuates NF-κB activation in TIS, confirming their interdependence in SASP regulation [109].

Mechanistically, TLR2 amplifies immunity-related SASP components during OIS through cGAS–STING coordination, while elevated LINE1 retrotransposons in late-stage senescence sustain interferon signaling via cytoplasmic cDNA-mediated pathway activation [72,113]. Recent findings have revealed that mitochondrial outer membrane permeabilization (miMOMP) enables BAX/BAK-mediated inner membrane herniation, releasing mitochondrial DNA (mtDNA) into the cytosol to trigger cGAS–STING-dependent SASP production [114]. Notably, mtDNA-driven cGAS–STING activation not only orchestrates SASP but also propagates age-associated chronic inflammation, establishing this pathway as a central integrator of senescence-related inflammatory networks [107,108,109,115].

## 6. Physiological Features of SnCs in the Eye

The global aging population is growing rapidly, and many age-related diseases are emerging alongside this demographic shift. The persistent accumulation of SnCs, driven by chronic stress, is a hallmark of aging and a key contributor to age-related diseases [116]. Similarly to other mature tissues, ocular senescence can be initiated by diverse external and internal stimuli (Table 1).

Ocular pathologies associated with aging, with onset typically occurring in the sixth decade of life, arise through the multifactorial interplay of core aging mechanisms encompassing proteostatic dysregulation, genetic predispositions, and cumulative environmental exposures. As individuals advance in age, the accumulation of detrimental stressors may precipitate pathological alterations, including the sprouting of neuronal dendrites and the activation of glial cells [117,118]. These changes can culminate in retinal degeneration and heighten the risk of visual impairment [119]. Among the age-related retinal disorders, AMD, DR, and glaucoma stand out as the predominant pathologies, often encountered in conjunction with cataracts [120]. In addition to having an impact on the retinal system, the increase in age also has a certain impact on the ocular surface and can lead to dry eye disease (Table 2).

As one of the most metabolically active tissues in the human body, the retina requires uninterrupted delivery of oxygen and essential nutrients to sustain its complex architecture and ensure proper physiological functioning [121,122]. Impaired angiogenesis and prolonged disturbances in oxygen and nutrient supply can trigger stress responses in essential retinal tissues, ultimately promoting cellular senescence [123]. The detection of pathognomonic signatures associated with cellular senescence, including upregulated cell cycle checkpoint regulators (p53, p16INK4a, and Cdkn1a/p21^CIP/WAF1) along with stress response mediators (PAI-1 and PML nuclear bodies), correlates with the accumulation of senescent cell populations across multiple retinopathy subtypes [122]. In ischemic retinopathy, neural and vascular cells exhibit varied responses to hypoxia and oxidative stress across retinal layers. At postnatal day 14 (P14), senescence initially appears in avascular areas before extending to pathological retinal microglia and vascular tufts [122]. Interestingly, apoptosis occurs more frequently in the inner nuclear layer (INL), while retinal ganglion cells (RGCs), responsible for transmitting visual signals to the brain, primarily display a senescent phenotype [122]. In primary open-angle glaucoma (POAG), senescence markers are detected in the trabecular meshwork. Aging of these cells impairs their function, reducing aqueous humor outflow and contributing to increased intraocular pressure (IOP) [124]. This age-related decline in outflow is also associated with a reduced population of RGCs [124]. In a mouse model of acute glaucoma, p53, a critical factor in cellular senescence, plays a role in RGC death, as demonstrated by the protection of RGCs in p53-deficient mice [23,125]. Moreover, experimental models of glaucoma-induced retinal damage revealed significant SASP activation, demonstrating that ocular hypertension triggers senescence-related inflammatory signaling in retinal tissues [125].

Optic nerve glial cells, primarily astrocytes and, to a lesser extent, microglia, undergo significant changes, including hyperreactivity and remodeling, which create an inflammatory environment that promotes RGC axonal death [126,127]. Reactive astrocytes are classified into two subtypes: A1, which is associated with neural ischemia and the upregulation of neurotoxins and complement genes, and A2, which is linked to neural inflammation and the upregulation of neurotrophic factors [126,128,129]. These reactive astrocytes secrete toxins that impose stress on RGCs, stimulate glial activation, and initiate a molecular cascade involving proteins like TGF-β1, TNF, CASP3, and p53 [126,130,131], potentially contributing to glaucoma and age-related RGC loss [132,133]. The factors influencing microglial phenotypes may stem from the aging retinal environment or intrinsic age-related changes in microglia, such as the accumulation of lipofuscin, which can enhance microglial activation and alter complement gene expression, thereby driving the emergence of pathogenic microglial phenotypes [134]. Additionally, retinal glial cells exhibit age-related changes, with aging leading to the activation of astrocytes and Müller glia, as evidenced by increased GFAP immunoreactivity, thicker cell layers, and the extension of cell processes [119].

Aging is a key contributor to the development of AMD, with its prevalence increasing significantly in individuals over 60. As the body ages, the retina undergoes structural and physiological changes, including a reduction in retinal neurons and accumulation of lipofuscin in the RPE, which can generate reactive oxygen species (ROS) when exposed to light and oxygen [135]. Cellular senescence, characterized by the upregulation of markers such as p16, p21, and p53, occurs in retinal neurons but not in blood vessels or neuroglia, leading to the loss of rod photoreceptors, RGCs, and rod bipolar cells [120]. Genetic factors also modulate susceptibility to retinal aging and degeneration. For instance, the SIX6 risk variant has been demonstrated to increase p16/INK4A levels, leading to RGC senescence, while amyloid-beta (Aβ), which accumulates in the aging retina and is a constituent of drusen, can trigger senescence in RPE cells [136]. While rare pathogenic variants (minor allele frequency [MAF] < 0.1%), such as those in WRN (OMIM 277700), follow Mendelian inheritance patterns, AMD is associated with common variants like CFH rs1061170 (MAF ~35%), which exhibit higher allele frequencies and necessitate the integration of polygenic risk scores for accurate risk prediction and clinical interpretation [137]. These findings collectively highlight the interplay between aging, genetic predisposition, and cellular senescence in the pathogenesis of AMD and underscore the therapeutic potential of targeting senescent retinal neurons and RPE cells in age-related retinal diseases.

Currently, the mechanisms underlying the heterogeneity of retinal cell senescence and its microenvironmental regulatory network have not been fully elucidated. In the future, it is necessary to integrate single-cell multi-omics, high-resolution spatiotemporal analysis, and gene-editing technologies to identify key nodal molecules and develop specific intervention strategies for human pathologies. Meanwhile, attention should be paid to the dynamic balance between senescence and regeneration (such as the reprogramming of Müller cells). It is also essential to explore a new therapeutic paradigm that reverses the senescent phenotype rather than simply eliminating senescent cells.

**Table 2:** Cellular senescence in ocular diseases and therapeutic strategies.

Disease	Senescent Cell Type/Mechanism	Key Senescence Markers	Representative SASP Components	Emerging Therapeutic Implications	References
DR	Endothelial cell senescence in retinal microvasculature; SASP-driven vascular dysfunction	p16INK4a, p21, p53, IGFBP3	IL-6, IL-1β, VEGF, MCP-1	Senolytics (UBX1325), cGAS–STING inhibitors, antioxidants, IL-6 trans-signaling blockade	[138,139]
AMD	Senescence of RPE cells; SASP-driven inflammation and drusen accumulation	p16, p21, p53, BMP4	IL-6, IL-8, MMPs, VEGF, complement proteins	Senolytics, autophagy enhancers, Piezo1-BMP4 inhibitors, PGAM5 modulators	[64,140]
Glaucoma	Senescence in trabecular meshwork and RGCs; increased outflow resistance leads to elevated intraocular pressure	p53, p21, p16, TGF-β1	IL-6, IL-8, MMP-9, TNF-α	Senolytics (Dasatinib, Quercetin), mTOR inhibitors, mitochondrial antioxidants, OSK factors	[65,141]
Cataracts	Senescence in lens epithelial cells; autophagy dysfunction and oxidative stress-induced protein aggregation	p21, LC3, p62	IL-6, ROS	HO-1/TFEB axis activators, autophagy modulators, antioxidant therapy	[142]
DED	Senescence-associated inflammation in lacrimal and meibomian glands; SASP-driven immune dysregulation	p16, 8-OHdG, SASP cytokines	IL-1β, MMP-3, MMP-9, IL-6	Senomorphic eye drops, mesenchymal stem cell therapy, mTOR inhibitors, mitochondrial transfer	[66]
Autoimmune/Inflammatory Eye Diseases	Senescence-associated immune dysfunction contributes to chronic inflammation in autoimmune uveitis	p16, p21, IFN-γ, IL-17A	GM-CSF, IL-17, IFN-γ, TNF-α	JAK-STAT inhibitors, immunosenescence modulators, Foxp3+ Treg reprogramming	[143,144]

Abbreviations: SASP—senescence-associated secretory phenotype; IL—interleukin; MMPs—matrix metalloproteinases; RPE—retinal pigment epithelium.

## 7. Mechanistic Insights into Cellular Senescence in Ocular Diseases

### 7.1. Diabetic Retinal Disease

DR, a leading cause of blindness in working-age adults globally [145], is characterized by progressive neurovascular degeneration in the retina [146,147]. The retina’s high metabolic demands and vascular complexity render it vulnerable to diabetes-induced metabolic disturbances, particularly through endothelial barrier dysfunction seen in diabetic macular edema (DME) [148]. Accumulating evidence positions cellular senescence as a central pathogenic mechanism in DR, with significant accumulation of SnCs observed in both clinical specimens and experimental models of diabetic retinopathy [122,139,149,150,151,152,153].

The cGAS–STING axis emerges as a critical senescence–inflammatory nexus in DR pathogenesis [138]. Diabetic retinas demonstrate coordinated upregulation of STING and senescence markers (p16INK4a, p21, p53, and Igfbp3), particularly within retinal endothelial cells (RECs) of proliferative DR patients and streptozotocin-induced models [138,153,154]. Mechanistically, STING activation via its canonical TBK1/IRF3/IFN-β axis not only amplifies DNA damage-driven senescence through type I interferon responses [107,155,156,157,158] but also directly compromises REC barrier integrity, providing a molecular bridge between cellular aging and DME-associated vascular pathology [138].

Current studies emphasize the cGAS–STING axis in DR-linked endothelial senescence but overlook cell-type-specific triggers (e.g., mitochondrial vs. nuclear DNA damage) and fail to address how obesity-linked inflammation in type 2 diabetes may synergistically amplify STING activation, a gap unmodeled by STZ-induced diabetic models. Hyperglycemia may drive vascular-specific senescence via mtDNA leakage in retinal endothelial cells, while neurons evade this through PINK1/Parkin-mediated mitophagy, suggesting that selective targeting of the non-canonical STING–STAT3–VEGF axis could mitigate DME without compromising systemic antiviral defenses.

### 7.2. Age-Related Macular Degeneration (AMD)

AMD, a leading cause of irreversible vision loss in individuals over 55 years, currently affects approximately 200 million people globally and is projected to reach 288 million cases by 2040 [64,159,160]. This complex neurodegenerative condition with multifactorial etiology presents two pathognomonic characteristics: (1) subretinal extracellular drusen formation containing aggregated lipid–protein–mineral complexes, and (2) cumulative deterioration of RPE cells accompanied by photoreceptor cell atrophy [161]. Emerging evidence suggests that vascular dysfunction is crucial to AMD pathogenesis, with early choroidal changes including endothelial cell loss in the choriocapillaris, reduced vascular density, and subsequent neovascularization that disrupts Bruch’s membrane [162,163,164]. These pathological alterations ultimately lead to fibrotic scarring and RPE detachment, contributing to central vision loss [163,164].

Beyond vascular alterations, cellular senescence has emerged as a critical mechanism in AMD progression, particularly affecting RPE and vascular endothelial cells. Age-related accumulation of senescent RPE cells, characterized by upregulated senescence markers (p16INK4A, p21CIP1, p53, and BMP4) and morphological alterations, correlates with drusen formation and macular degeneration [165,166,167]. The functional decline of aging RPE compromises the outer blood–retinal barrier, creating a permissive environment for oxidative damage and mitochondrial dysfunction [168,169]. While mitochondrial DNA damage is recognized in AMD patients, the relative contributions of mitochondrial versus nuclear DNA damage responses remain unclear [169].

Recent studies have implicated PGAM5, a mitochondrial phosphatase regulating fission through DRP1 dephosphorylation, as a novel modulator of AMD-related senescence [140,170,171]. PGAM5 deficiency in murine models induces mitochondrial hyperfusion, elevated reactive oxygen/nitrogen species (RONS), and accelerated RPE senescence through mTOR and IRF/IFN-β pathway activation [140]. Paradoxically, PGAM5 knockout confers protection against acute oxidative stress in young mice, suggesting age-dependent effects on RPE survival [140]. These findings position PGAM5 at the intersection of mitochondrial dynamics, oxidative stress, and senescence—key pathways in AMD pathogenesis. Although PGAM5 deficiency is linked to mitochondrial hyperfusion and RPE senescence acceleration, current studies overlook its age-dependent paradox: protective in youth, but degenerative in aging. Moreover, whether this duality results from changes in interacting partners (e.g., mTOR vs. IRF/IFN-β) or tissue-specific redox thresholds remains unclear.

In addition, drusen-mediated mechanical stress can collaborate with RPE senescence. By activating mechanosensitive ion channels such as Piezo1, it triggers calcium-dependent BMP4/SMAD signaling, which spreads senescence laterally. Targeting the Piezo1-BMP4 cross-talk offers a potential way to break this feedforward loop while maintaining normal mitochondrial fission. The SASP plays a central part in AMD progression, although its manifestations vary among cell types. Post-mitotic photoreceptors and proliferative RPE cells likely undergo senescence through different mechanisms, yet both contribute to degenerative processes via SASP-mediated inflammation and tissue remodeling [140]. Given the complex and cell-type-specific nature of these mechanisms, therapeutic strategies must be precisely tailored to the underlying senescent pathways. Representative approaches and clinical applications are further discussed in Section 8.

### 7.3. Glaucoma

Glaucoma is the leading cause of irreversible blindness globally. Around 3.5% of people aged 40 to 80 have glaucoma. As the elderly population grows, by 2040, an estimated 111.8 million people will have glaucoma [172]. This condition is characterized by progressive degeneration of RGCs and their axons, culminating in visual impairment. While elevated IOP and aging remain primary risk factors [65,173], genome-wide association studies (GWASs) have identified several common susceptibility loci associated with POAG, including SIX1–SIX6 and CDKN2A/B (p16^INK4A^) [174,175].These variants confer modest risk and contribute to disease susceptibility in the general population, in contrast to rare, high-penetrance mutations such as those in Myocilin (MYOC) and optineurin (OPTN), which underlie monogenic, early-onset forms of glaucoma [176,177].

Emerging evidence implicates autophagy dysregulation in glaucoma pathogenesis. Clinical studies have revealed reduced autophagic activity in glaucoma patients, particularly those with exfoliation syndrome [178,179,180]. Paradoxically, murine models have demonstrated both protective and detrimental roles of autophagy. While enhancing mitophagy shows neuroprotective effects in glaucoma models by clearing dysfunctional mitochondria [181,182], pharmacological autophagy inhibition with 3-MA paradoxically reduces RGC death [183]. Age-related declines in proteostasis may exacerbate this dichotomy, as demonstrated by Ambra1+/gt mice studies showing impaired Nrf2/Bnip signaling and increased oxidative stress in aged mutants compared to younger counterparts [184].

The autophagy–ubiquitin axis appears particularly relevant to glaucoma pathology. Mutations in autophagy-related proteins TBK1 and OPTN—both involved in cargo recognition through ubiquitin-LC3 interactions—have been linked to disease [185]. Notably, excessive activation of these proteins induces apoptotic cell death in vitro [186]. Concurrently, ubiquitin–proteasome system (UPS) dysfunction emerges as a contributing factor. Comparative analyses of pseudoexfoliation syndrome patients have revealed decreased ubiquitin expression, reduced proteasome activity, and elevated ER stress compared to healthy controls [187]. These findings align with observations in DBA/2J glaucoma mice showing age-dependent reductions in retinal ubiquitin levels and accumulation of ubiquitinated mitofusin 2 in RGCs [181].

The interplay between autophagy and UPS dysfunction may create a pathological feedback loop in glaucoma. Impaired autophagic flux in hypertensive DBA/2J mice correlates with both disease progression and accumulation of ubiquitinated proteins [188]. This dual failure of cellular clearance mechanisms likely exacerbates mitochondrial dysfunction and oxidative stress, ultimately driving RGC degeneration.

### 7.4. Age-Related Cataract

Age-related cataracts represent the predominant cause of global blindness, primarily affecting individuals beyond 50 years through progressive lens opacification [189]. Central to this pathology are lens epithelial cells (LECs), which regulate the lens microenvironment critical for optical clarity [190]. Aging induces progressive LEC depletion (~14% over 75 years), accelerated by oxidative stress that triggers cataractogenesis when surpassing cellular compensatory capacity [190,191,192]. The mechanistic cascade involves oxidative stress-mediated LEC apoptosis and autophagic dysregulation—sustained autophagic activity during cellular aging promotes p62 scaffold protein degradation, disrupting pro-survival signaling and precipitating premature senescence [193]. Pharmacological autophagy inhibition demonstrates therapeutic potential by preserving p62 levels and attenuating apoptotic pathways [193], highlighting the need to elucidate autophagy’s dual roles in LEC homeostasis and cataract pathogenesis.

The disease complexity extends to protein homeostasis alterations. Compromised autophagy and crystallin modifications (mutations/aggregations) synergistically drive cataract formation through accumulation of oxidized proteins and insoluble crystallin complexes [194,195]. The “cataractogenic load” hypothesis posits age-dependent accumulation of molecular stressors affecting lens biophysics (size, rigidity, and refractive properties) and biochemistry (membrane-bound α-crystallin chaperone dysregulation) [142,196,197]. These cumulative changes disrupt the lens’ capacity to manage denatured proteins, with protein aggregation emerging as both a biomarker and effector of ocular aging pathology.

While oxidative stress and autophagic dysregulation are well-established contributors to LEC senescence and cataractogenesis, the precise balance between autophagy’s protective and detrimental roles remains incompletely understood. Notably, while pharmacological autophagy inhibition has been shown to preserve p62 and mitigate apoptosis, excessive autophagy suppression may impair cellular proteostasis, exacerbating crystallin aggregation and lens opacity. Therefore, future studies should focus on delineating the threshold at which autophagy transitions from a protective mechanism to a pathogenic driver, enabling the development of targeted interventions that fine-tune autophagic flux without compromising lens homeostasis.

### 7.5. Dry Eye Disease

The ocular surface is essential for protecting the eye’s internal structures and maintaining corneal smoothness through the tear film, which is critical for optimal vision. However, aging enhances the ocular surface’s susceptibility to external factors, contributing to conditions such as DED, a prevalent age-related disorder marked by tear film instability, ocular surface inflammation, and neurosensory dysfunction [198,199]. With age, tear volume decreases, tear film breakup time shortens, and tear osmolarity increases [200,201,202,203,204,205,206], along with elevated levels of pro-inflammatory cytokines and a decrease in insulin-like growth factor (IGF)-1, which is significantly associated with DED. The proportion and quality of immune cell populations at the ocular surface change as we age [207,208], potentially linking cellular senescence to the decline in immune function and the pathology of DED. Age-related changes in the lacrimal gland include reduced acinar density, increased inflammatory cell infiltration, and elevated levels of oxidative stress markers such as 8-hydroxydeoxyguanosine (8-OHdG), contributing to DED [209,210]. Senescence-associated T (SA-T) cells and additional immune cells infiltrate the lacrimal glands of aged mice, indicating that cellular senescence could contribute to the pathology of DED [211].

Age-related changes also impact the meibomian glands, affecting the expression of peroxisome proliferator-activated receptor (PPAR)-γ in meibocytes [212], regulated by dickkopf-like acrosomal protein 1 (Dkkl1) [213], leading to alterations in meibum’s hydrocarbon chain order and phase transition temperatures [214]. These changes may contribute to the age-related alterations in the lipid composition of human meibum [215]. Meibocytes in older mice show reduced expression of cytokeratin 1, possibly contributing to the anterior migration of the mucocutaneous junction (MCJ) [216]. Aging induces significant changes in the meibomian glands that impair their function, including gland dropout, orifice plugging, cystic dilation, hyperkeratinization of the ducts, and modifications in meibum composition [217,218]. Meibomian gland dysfunction is prevalent in the aging population and plays a key role in the vicious cycle of dry eye by contributing to tear film instability and increasing tear hyperosmolarity [217].

Cellular senescence likely contributes to lacrimal and meibomian gland dysfunction in age-related DED. However, it is uncertain whether SASP factors directly inhibit lipid synthesis in meibocytes or indirectly affect tear secretion. The interaction between senescent gland epithelia and ocular microbiota is also uninvestigated. To advance beyond symptomatic treatment, future research should focus on single-cell transcriptomic analysis, targeted senomorphic therapies like IL-1α/β-neutralizing microparticles, and mitochondrial transfer for meibocyte function restoration.

### 7.6. Autoimmune and Inflammatory Eye Diseases

Autoimmune uveitis (AU), an inflammatory disorder targeting the central nervous system (CNS), manifests as immune-mediated ocular damage and is a leading cause of acquired blindness [219,220]. Mechanistic insights into AU pathogenesis derive principally from experimental autoimmune uveitis (EAU) models, which recapitulate key immunopathological features of human disease [221].

Lymph nodes (LNs), as peripheral immune hubs, facilitate coordinated immune cell interactions essential for adaptive immunity and pathogen surveillance [222]. Age-related lymphoid tissue remodeling involves structural disorganization (e.g., fibroblastic reticular cell network fragmentation), impaired chemokine-guided lymphocyte trafficking, and attenuated germinal center reactions [223,224]. Intriguingly, aged murine EAU models have demonstrated mitigated disease progression through Th17 cell hyporesponsiveness, suggesting age-dependent immunomodulation of autoimmune pathways [225]. Paradoxically, senescence may concurrently prime systemic autoimmunity via bystander activation of autoreactive lymphocytes, warranting further mechanistic dissection.

A key study on aging’s effect on ocular health found that NOD.B10.H2b mice showed increased susceptibility to Sjögren syndrome (SS) and non-SS keratoconjunctivitis sicca (KCS) with age [226]. The aged mice had increased corneal permeability, CD4+ T cell infiltration, and loss of conjunctival goblet cells, along with lacrimal gland atrophy and higher levels of inflammatory cytokines [226]. Notably, with age, there is an increase in the frequency of CD4+Foxp3+ Tregs, which paradoxically lose their suppressive functions and begin to produce IL-17 and IFN-γ [226]. This shift suggests a role for these cells in the pathogenesis of age-related ocular surface diseases, contributing to conditions such as lacrimal keratoconjunctivitis [226].

In summary, diverse in vitro and in vivo models have demonstrated that cellular senescence contributes to structural and functional damage in the retina, lens, trabecular meshwork, and ocular surface. These experimental findings establish a mechanistic foundation for the development of senescence-targeting therapies, which are further discussed in the next section with a focus on clinical translation.

## 8. Clinical Applications of Senescence-Targeting Strategies in Ocular Diseases

Current research priorities emphasize elucidating the pathological contributions of cellular senescence, with the ultimate objective of transforming this biological process into a therapeutically targetable mechanism [227]. The clinical relevance of cellular senescence has spurred investigations into therapeutic approaches aimed at SnCs. Extensive research has shown that senotherapeutics can prolong a healthy lifespan and mitigate age-related disorders. Broadly, senotherapeutics fall into two groups: (1) senolytics, which specifically target and remove SnCs, and (2) senomorphics, which modify the harmful effects of the SASP [5]. Ongoing research and clinical trials suggest these agents may restore tissue homeostasis, suppress inflammation, and promote ocular tissue regeneration.

### 8.1. Senolytics

Senolytics have emerged as a promising class of drugs in ocular aging therapy [29]. Unlike traditional strategies that inhibit aging pathways (e.g., p53, p16, and p21) and pose potential oncogenic risks, senolytics selectively eliminate SnCs, offering a safer and more targeted approach [228,229,230]. The first identified senolytics, dasatinib and quercetin, were shown to effectively reduce senescent cell burden and alleviate age-related symptoms in animal models [231]. These results highlighted the potential of senolytics to extend health span. Following these discoveries, other drugs, such as navitoclax, 17-DMAG, and peptides targeting Bcl-2 and p53-related pathways, further confirmed the benefits of selectively targeting SnCs [232,233].

Navitoclax (ABT-263), a Bcl-2 family protein inhibitor, emerged as a powerful senolytic, binding selectively to B cell lymphoma-extra large (Bcl-xL), Bcl-2, and Bcl-w to prevent their interaction with apoptotic effectors [234,235,236]. A new category of senolytic agents targeting heat-shock protein 90 (HSP90) has also been identified [237]. For instance, the HSP90 inhibitor 17-DMAG markedly delayed age-related symptoms in progeroid mice, enhancing their overall health span [237]. Another recently identified senolytic agent, piperlongumine (PL), selectively targets SnCs by interacting with oxidation resistance 1 (OXR1), enhancing its senolytic activity [238].

Emerging studies also reveal that cytosolic DNA activates the cGAS–STING pathway, triggering inflammation and contributing to senescence. Inhibiting this pathway may provide a therapeutic strategy for preventing senescence-associated diseases. Moreover, bromodomain and extraterminal (BET) family protein degraders (BETd) trigger senolysis via two linked mechanisms: suppression of non-homologous end joining (NHEJ) and induction of autophagy [53]. BETd also reduce senescent hematopoietic stem cells in tumor microenvironments, showing promise for cancer prevention [53].

Immunotherapy targeting SnCs is gaining attention as an alternative to directly eliminating them with external drugs. This approach boosts immune-mediated clearance of SnCs, advocating for a balanced intervention. Strategies like ICOS-activating antibodies enhance senescence surveillance, while combining senolytics with immune-mediated clearance can synergistically reduce senescent cell numbers [239]. Additionally, emerging immunotherapeutic strategies focus on targeted depletion of senescence-associated surface antigens, exemplified by chimeric antigen receptor T cell-mediated clearance and PD-L1+ cell elimination, demonstrating significant therapeutic potential [240,241].

### 8.2. Senomorphics

Although senolytic interventions show therapeutic potential, their application risks disrupting senescence-associated tumor surveillance mechanisms and impairing physiologically beneficial aspects of cellular aging. An alternative approach is targeting the pro-inflammatory microenvironment and suppressing the SASP, which could provide a safer route for senotherapy [242,243]. In contrast to senolytics, which specifically eliminate SnCs, senomorphic agents focus on modulating pro-inflammatory signaling pathways [244]. Several FDA-approved drugs, such as neutralizing antibodies against IL-1α or its receptor and mTOR inhibitors like rapamycin, can modulate these pathways and suppress SASP activity [39,245]. While anti-inflammatory treatments show promise, they can lead to side effects. Therefore, targeting specific SASP components may provide a safer alternative [246]. Monoclonal antibodies can neutralize cytokines like IL-6, which are key drivers of the SASP. FDA-approved drugs like siltuximab and tocilizumab, which block IL-6 or its receptor, are already used for cytokine release syndrome and IL-6-driven diseases and could be repurposed to manage senescence [246].

Research into senescence mechanisms in ocular cells is still developing, with clinical trials for senolytic drugs in age-related eye diseases like macular degeneration underway [247]. UBX-1967, a Bcl-2 family inhibitor, shows promise for treating neovascular AMD and DR [247]. UBX-1325, targeting BCL-xL, is another potential treatment for age-related retinal disorders. Our team has identified procyanidin C1 (PCC1), a compound from grape seed extract, as a promising alternative to traditional drugs [119]. PCC1 reduces SnCs, suppresses SASP, and restores age-related gene regulation, potentially slowing retinal disease progression [119]. In DR models in vitro, PCC1 reduced senescence and inflammatory markers, suggesting that it offers superior safety, bioavailability, and efficacy compared to other senolytics [119]. Although the outcomes of these studies are encouraging, their long-term success hinges on a more comprehensive understanding of the mechanisms underlying ocular disease progression, which is essential for designing effective therapies that address the root cause of retinal dysfunction.

### 8.3. Treatment Strategies for Diabetes-Related Retinopathy

Clinical advances in DR are beginning to leverage the therapeutic potential of senescence-targeting strategies. Hyperglycemia-induced senescence in retinal vascular endothelial cells has been implicated in impaired barrier function and persistent inflammation [248]. Rather than focusing solely on the mechanistic underpinnings, current efforts emphasize the clinical translation of senolytics and senomorphics to counteract these pathological changes.

Senolytic therapies targeting BCL-xL demonstrate promising clinical translation in retinal diseases [153]. UBX1325, a senolytic drug that induces senolysis by inhibiting BCL-xL, has shown promise in promoting vascular repair and remodeling in diabetic retinas [248]. Intravitreal administration of UBX1325 targets and removes senescent endothelial cells, reducing inflammation and enhancing vascular barrier function, which can improve vision [248]. In a phase 2b clinical trial for DME patients, a single administration of UBX1325 led to a statistically significant and clinically relevant improvement in mean best corrected visual acuity (BCVA) over 48 weeks, compared to placebo treatment. UBX1325 has shown a positive safety and tolerability profile, with no signs of intraocular inflammation, indicating its potential as a long-lasting, disease-altering treatment for DME, although further large-scale clinical trials are needed to confirm this hypothesis [248]. UBX1967, another BCL-xL-targeting agent, is under investigation for proliferative retinopathy and has demonstrated efficacy in promoting regression of pathological neovascularization in preclinical models [153].

Researchers have highlighted that IL-6 trans-signaling (TS) plays a significant role in inflammation and vascular dysfunction in DR [249,250,251], leading to mitochondrial dysfunction and increased superoxide production in RECs [251]. Hoffman, J.M. et al. further demonstrated that targeting IL-6 TS with sgp130Fc could be an effective anti-inflammatory strategy for DR and possibly other inflammatory diseases [252]. Further in vivo research is needed to explore the impact of IL-6 TS in age-related diseases and its potential as a therapeutic target for delaying aging and improving overall health.

Overall, the integration of senescence-targeting therapies in DR treatment strategies—particularly through clinical-stage compounds like UBX1325—represents a shift toward regenerative and precision medicine in managing diabetic retinal complications.

### 8.4. Treatment Strategies for AMD

Senescence-targeting strategies are emerging as a promising approach for treating AMD, focusing on eliminating SnCs, suppressing pro-inflammatory SASP factors, and restoring RPE function. Senolytic small molecules, such as UBX1325 and UBX1967, target SnCs by inducing apoptosis, showing promise for AMD treatment. Although initially studied in diabetic eye disease, UBX1325 is undergoing early-phase clinical trials, and its pharmacokinetic and safety data will inform its application in AMD [29,248]. High-throughput screening has identified small compounds, including BET protein inhibitors, which reduce RPE cell reduction in vitro and protect RGCs in vivo [253,254]. These compounds may be repositioned for AMD treatment, pending further clinical validation.

Oxidative stress-induced cellular senescence is a critical contributor to AMD pathogenesis [255]. BMP4, a TGF-β superfamily member, regulates RPE proliferation and morphogenesis. Elevated BMP4 levels in oxidative stress-exposed RPE cells and dry AMD patients induce senescence via Smad, p38, and p53/p21CIP1 pathways, alongside reduced RB phosphorylation [256,257]. BMP4 antagonists, including Chordin-like 1 protein and the p38 inhibitor SB203580, counteract premature RPE senescence [246]. Sumoylation, a reversible post-translational modification involving small ubiquitin-like modifiers (SUMOs), regulates retinal and RPE aging by modulating proteins like p53 and RB. In retinal pigment epithelial cells, inhibition of sumoylation modification results in reduced inflammatory cytokine expression during oxidative stress-associated cellular aging [258,259].

Additional therapeutic candidates are targeting mitochondrial dysfunction and oxidative damage in the aging retina. Agents such as Humanin and Elovanoids (ELVs) have shown protective effects by enhancing mitochondrial function and reducing senescence-induced inflammation [260,261,262]. These biologics, while still in the preclinical phase, represent innovative directions in age-related retinal therapy.

Retina-resident macrophages, affected by aging and senescence, contribute to AMD pathogenesis through altered polarization and pro-inflammatory secretion [263,264,265,266]. LXR agonists and miR-33 inhibitors have demonstrated the capacity to restore cholesterol homeostasis and reduce retinal inflammation in animal models [267], supporting their potential in clinical translation.

An overview of current AMD therapies, such as cell replacement treatments and ongoing clinical trials, can be found in other sources [268,269,270,271,272]. Emerging strategies such as partial epigenetic reprogramming and statin therapy are being evaluated alongside senotherapeutics [273]. While AREDS-recommended supplements remain a mainstay for patients with geographic atrophy, novel senescence-targeting therapies could redefine disease-modifying treatment approaches for AMD in the near future.

### 8.5. Treatment Strategies for Glaucoma

There is increasing interest in therapies that delay cellular senescence, given its association with aging and glaucoma, as senescence accelerates in this condition. Treatments targeting specific pathways of premature senescence are under development. Current agents, such as antioxidants, autophagy enhancers, and DNA repair stimulators, can help reduce premature senescence by modulating ROS levels and oxidative DNA damage [274]. Selective removal of SnCs also offers a promising therapeutic approach to prevent, delay, or mitigate glaucoma [274].

Neurodegenerative disorders, including glaucoma, have shown promising responses to antioxidant therapies in both cellular and animal models [275]. Coenzyme Q10, a vital component of the electron transport chain, supports mitochondrial function by maintaining membrane potential, facilitating ATP synthesis, and protecting against free radical damage [276]. In glaucoma models, coenzyme Q10 enhances RGC survival by preserving mtDNA content and upregulating oxidative phosphorylation complex IV proteins and mitochondrial transcription factor A [277]. Similarly, oral vitamin B3 supplementation and gene therapy designed to enhance NAD+ levels have demonstrated protective effects in aging mice, suggesting potential for glaucoma prevention and treatment [278].

Targeted antioxidant delivery methods, such as intraocular implants or nanotechnology, may improve treatment efficacy. Mitochondria-specific antioxidants, which can penetrate mitochondrial membranes and neutralize ROS at their source, have shown greater effectiveness than nonspecific antioxidants [279]. For instance, MitoQ, a compound formed by conjugating coenzyme Q with triphenylphosphonium (TPP) [280], reduces age-related oxidative stress and supports healthy aging [281]. Another TPP-conjugated compound, SkQ1, has shown therapeutic potential in glaucoma models by lowering IOP and alleviating glaucoma-related symptoms [282]. However, additional studies are required to assess whether antioxidants can effectively prevent RGC death by mitigating the pro-aging effects of cellular senescence.

Emerging evidence highlights the role of mTOR inhibition in extending the lifespan and improving mitochondrial function by reducing oxidative stress [283,284]. Rapamycin, for example, activates REDD1 to inhibit mTOR signaling, enhancing mitochondrial function and rescuing dying RGCs [285]. Additionally, epigenetic reprogramming has shown potential in reversing senescence and repairing associated damage [286]. For instance, ectopic expression of Oct4, Sox2, and Klf4 (OSK) in mouse RGCs restored youthful DNA methylation patterns and transcriptomes, promoting axonal regeneration and reversing vision loss in glaucoma and aging models [287]. By utilizing a tetracycline-responsive dual adeno-associated virus system, researchers were able to reverse vision loss caused by glaucomatous injury, underscoring the promise of epigenetic rejuvenation as a potential approach to counteract senescence in glaucoma.

Targeting SnCs has emerged as a promising strategy to counteract aging-related diseases by preventing, delaying, or alleviating the senescence phenotype [231]. Drugs like dasatinib and quercetin have shown potential to preserve retinal structure under elevated intraocular pressure by selectively clearing SnCs [288,289,290]. Senolytics, by clearing SnCs, could help reduce the negative impact of elevated IOP on RGC survival in glaucoma and other optic neuropathies. While early studies have not always assessed senescence markers directly, the neuroprotective outcomes suggest these agents could play a role in mitigating glaucomatous damage [291]. Further clinical research is needed to validate their efficacy and safety in humans.

The combination of senescence-targeting agents—whether antioxidant, genetic, or senolytic—with existing neuroprotective measures may provide a comprehensive strategy to alter glaucoma progression. Continued investigation into these therapies in clinical settings will help refine their application and optimize treatment protocols for aging-related optic neuropathies.

### 8.6. Treatment Strategies for Cataract

Age-related nuclear cataract (ARNC) is increasingly being understood as a condition involving premature cellular senescence and impaired proteostasis in lens epithelial cells (LECs). Recent studies have identified the HO-1/TFEB signaling axis as a key regulatory pathway that may be therapeutically leveraged to counteract cataract progression. In preclinical models, HO-1 activation restored autophagic flux and lysosomal function in stressed LECs, reducing oxidative damage and delaying senescence [292].

These findings support the potential clinical translation of strategies combining antioxidants with lysosomal function modulators to preserve lens transparency. Rather than focusing solely on the underlying mechanisms, current efforts emphasize therapeutic applications, such as pharmacological activation of HO-1 or TFEB, to rejuvenate aged LECs [292]. This approach may complement or enhance existing cataract prevention strategies, offering an alternative to surgical intervention in early-stage disease.

Future research and clinical trials are warranted to validate the safety, dosing, and delivery of HO-1/TFEB-targeting agents in patients. As senescence-targeting therapies expand beyond retinal diseases, cataract represents a compelling frontier for innovative intervention aimed at maintaining lens clarity in the aging eye.

### 8.7. Treatment Strategies for Ocular Surface Diseases

Kojic acid, a natural microbial metabolite with established safety as a dermal depigmenting agent and food preservative, demonstrates multifaceted bioactivity including antioxidant, antitumor, and anti-inflammatory properties [3]. Recent mechanistic studies have revealed its therapeutic potential in corneal endothelial pathologies through dual suppression of NF-κB and p21 pathways, effectively counteracting cellular senescence and associated pathological angiogenesis [293]. These findings position this compound as a promising candidate for managing Fuchs’ endothelial dystrophy and related senescence-driven ocular disorders, though precise molecular targets within such natural products require further elucidation.

Parallel investigations have identified miR-30c-1 as a TGF-β antagonist that attenuates oxidative damage and SASP production in corneal cells [294], while the mTOR inhibitor rapamycin delays senescence progression while preserving epithelial barrier function through combined anti-apoptotic mechanisms [295]. Complementary preclinical evidence shows that ABT-263-mediated senolysis restores lacrimal gland homeostasis in murine graft-versus-host disease (GVHD) models [296].Although extensive senotherapeutic development focuses on systemic age-related diseases [242,244,297,298], these ocular findings provide critical proof-of-concept for adapting senescence-targeting strategies to visual system pathologies.

## 9. Conclusions

Cellular senescence plays a critical role in the pathogenesis of numerous age-related ocular diseases, including AMD, DR, glaucoma, cataracts, and ocular surface disorders. By driving chronic inflammation, oxidative stress, and tissue dysfunction through the SASP, SnCs contribute to the initiation and progression of these diseases. While the presence of SnCs may serve protective functions under specific conditions, their prolonged accumulation ultimately exerts detrimental effects on ocular health.

This study, from a unique perspective of cellular senescence, reveals the complex and intricate connections between cellular senescence and ocular diseases. It also identifies the key regulatory nodes of cellular senescence and novel mechanisms of action in multiple diseases, which have received relatively little attention in previous research. The development of therapeutic strategies targeting cellular senescence offers a promising avenue for addressing these age-related diseases. Approaches such as senolytics, SASP modulators, mitochondria-targeted antioxidants, and epigenetic reprogramming have shown encouraging results in preclinical models, demonstrating their potential to reduce senescent cell burden, restore tissue function, and slow disease progression. Furthermore, advances in delivery methods, including nanotechnology and viral vectors, hold great promise for optimizing the efficacy and safety of these treatments in ocular tissues.

However, significant challenges remain before these therapies can be translated into clinical practice. A more thorough understanding of the molecular mechanisms driving cellular senescence in various ocular diseases is essential, along with further research into the long-term safety and specificity of interventions targeting senescence. Additionally, the dual roles of SnCs—both protective and pathological—necessitate a careful balance between eliminating harmful cells and preserving their beneficial effects.

In summary, this study brings about a paradigm shift in the management of age-related ocular diseases from the perspective of cellular senescence. SnCs-targeted therapies, such as senolytics and senomorphics, function as regenerative interventions by suppressing senescence-associated inflammation, restoring tissue-specific physiological homeostasis, and directly promoting the regeneration of structural and functional ocular defects. By addressing the root causes of disease progression through these mechanisms, such regenerative therapies not only improve patient outcomes but also hold transformative potential for revolutionizing the ophthalmological treatment landscape. Future research should prioritize refining these strategies to enhance their precision and safety while further elucidating the interplay between senescence, aging, and ocular pathophysiology, ultimately enabling the development of clinically effective regenerative solutions.

## Figures and Tables

**Table 1:** Mechanisms and pathways of cellular senescence in ocular diseases.

Inducer	Senescence Pathway in Ocular Context
Oxidative stress	Chronic exposure to oxidative stress in RPE cells induces DNA damage and mitochondrial dysfunction, leading to p53–p21-mediated senescence.
Chronic inflammation	Prolonged inflammatory signaling in the retina or uveal tract activates NF-κB and cytokine secretion (e.g., IL-6, IL-8), reinforcing the SASP and promoting senescence.
Accumulation of metabolic waste	In diseases like AMD, impaired autophagy and accumulation of lipofuscin or drusen contribute to lysosomal stress, mitochondrial dysfunction, and subsequent senescence.
Light-induced damage	High-energy light exposure (e.g., blue light) causes photo-oxidative damage to RPE cells, triggering DNA damage response and activation of the p16–RB pathway.
Age-related epigenetic changes	Epigenetic drift with age (e.g., histone modification and DNA methylation changes) disrupts gene expression in retinal cells, promoting p16 or p21 expression and senescence.
Genetic alterations	*Rare pathogenic mutations:* High-penetrance mutations (e.g., *WRN* and *LMNA*) causing defective DNA repair in monogenic retinal diseases (e.g., retinitis pigmentosa) *Common risk variants:* GWAS-identified loci (e.g., *CFH* and *ARMS2* in AMD) with small effects (OR~1.2–3.0), interacting with environmental stressors.
Hypoxia or ischemia	Retinal ischemia induces oxidative stress and HIF-1α signaling, which may lead to endothelial cell senescence and neovascular dysfunction.

## Data Availability

Not applicable.

## Abbreviations

AMD	Age-related macular degeneration
AMPK	AMP-activated protein kinase
ARNC	Age-related nuclear cataract
AU	Autoimmune uveitis
BCL-xL	B cell lymphoma-extra large
BET	Bromodomain and extraterminal
BETd	BET family protein degraders
C/EBP	CCAAT/enhancer-binding protein
CCFs	Cytoplasmic chromatin fragments
CDKs	Cyclin-dependent kinases
cGAS–STING	Cyclic GMP-AMP synthase–stimulator of interferon genes
CNS	Central nervous system
DAMPs	Damage-associated molecular patterns
DBD	DNA-binding domain
DDR	DNA damage response
DED	Dry eye disease
DME	Diabetic macular edema
DPS	Developmentally programmed senescence
DR	Diabetic retinopathy
DSBs	Double-strand breaks
EAU	Experimental autoimmune uveitis
EMT	Epithelial-to-mesenchymal transition
EVs	Extracellular vesicles
FED	Fuchs’ endothelial dystrophy
GWAS	Genome-wide association studies
HCC	Hepatocellular carcinoma
HMGB1	High mobility group box 1 protein
HUVECs	Human umbilical vein endothelial cells
IGFBP	Insulin-like growth factor-binding proteins
INL	Inner nuclear layer
IOP	Intraocular pressure
LECs	Lens epithelial cells
LNs	Lymph nodes
MCJ	mucocutaneous junction
NHEJ	Non-homologous end joining
NK	Natural killer
OIS	Oncogene-induced senescence
PAI	Plasminogen activator inhibitor
PARP1	Polymerase 1
PCC1	Procyanidin C1
PDR	Proliferative diabetic retinopathy
PKD	Protein kinase D
PML	Promyelocytic leukemia protein
POAG	Primary open-angle glaucoma
RECs	Retinal endothelial cells
RGCs	Retinal ganglion cells
RONS	Reactive oxygen and nitrogen species
ROS	Reactive oxygen species
RPE	Retinal pigment epithelium
RS	Replicative senescence
SAHF	Senescence-associated heterochromatic foci
SASP	Senescence-associated secretory phenotype
SIPS	Stress-induced premature senescence
SnCs	Senescent cells
STING	Stimulator of interferon genes
TAD	Transactivation domain
TFEB	Transcription factor EB
TIMP	Tissue inhibitors of metalloproteases
TIS	Therapy-induced senescence
TOP1cc	Topoisomerase 1 cleavage complexes
TSPCs	Tendon stem/progenitor cells
UPS	Ubiquitin-proteasome system
VEGF	Vascular endothelial growth factor
